# Supplementary material for: TSProm: Deciphering the Genomic Context of Tissue Specificity
Source: bioRxiv. 2025 Oct 31:2025.10.30.685161. Preprint. [Version 1] doi: 10.1101/2025.10.30.685161 (PMC12636395; doi:10.1101/2025.10.30.685161)
Supplement: Supplement 1 [file media-1.pdf]

Appendix A. Tissue choice for `TSProm`

We selected testis, brain, and liver because these tissues contained the highest number of transcripts, from which we extracted regions around the transcription start site (TSS). To address reviewer concerns about the applicability of `TSProm` to other tissues, we extended the analysis to two additional tissue transcript sets: muscle and spleen. Ovary was not included since the number of transcripts in the human data was less than 100, which was insufficient for robust training. We aimed to have at least 1,000 transcripts in the positive set to reduce the risk of overfitting for these large language models.

We used `MMseqs2` to remove sequences with more than 80% similarity, which reduced the number of unique transcripts available for each tissue. The final datasets were divided into training, validation, and test subsets to ensure balanced evaluation.

Table 1: Transcript counts by tissue and source in mouse and human `TransTEx` datasets.

**Tissue**	**Mouse TransTEx**	**Human TransTEx**	**Human + Mouse**
Testis	9,045	12,459	21,504
Brain	2,493	612	3,105
Liver	427	774	1,201
Ovary	1,032	71	1,103
Muscle	264	338	602
Spleen	348	187	535
Lung	242	142	384
Bone marrow, blood, vessels	156	204	360
Heart	219	126	345
Adrenal gland	150	117	267
Small intestine	143	57	200
Vesicular / salivary gland	10	76	86
Stomach	50	2	52
**Total (13 common tissues)**	14,579	15,165	—

Totals mapped to Ensembl gene names: Mouse TransTEx: 17,320; Human TransTEx: 17,619.

Appendix B. Rationale for sequence length and TSS-centered regions

Transcription factor binding sites (TFBS) located near the transcription start site (TSS) typically exert the strongest influence on transcriptional activity. These sites can act as activators or repressors depending on the transcription factor and its interaction with other cofactors. Although more distal TFBS also play roles in gene regulation, their effects are generally mediated through cooperative interactions or chromatin looping.

To determine the most biologically meaningful sequence length around the TSS, we analyzed transcription factor binding data and histone mark profiles from the ENCODE consortium. The enrichment of TFBS around the TSS indicates that most experimentally validated sites occur within approximately 3 kilobases upstream and 1 kilobase downstream of the TSS. This range represents a core promoter-proximal region where transcriptional regulation is most direct, while still encompassing some distal elements that contribute to gene-specific expression (Georgakopoulos-Soares et al., 2023; Tharakaraman et al., 2008; Veerla and Höglund, 2006).

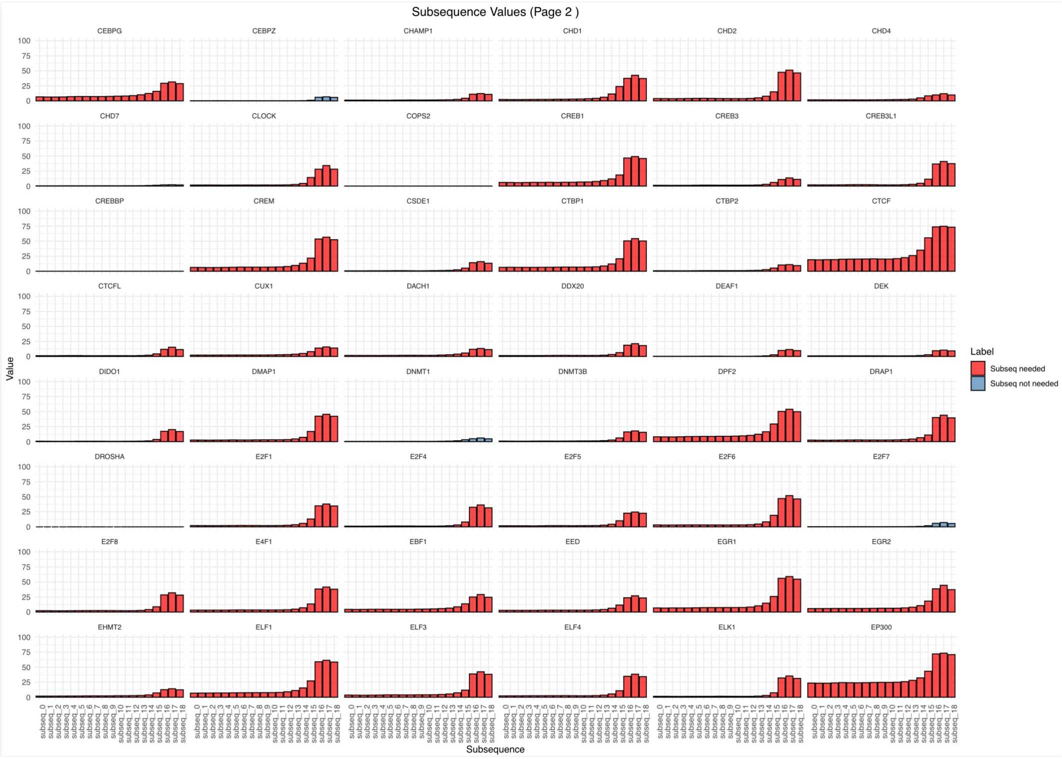

Figure 1: Illustrative example showing the enrichment of transcription factor binding sites (TFBS) near the transcription start site (TSS). The highest density occurs within ∼3 kb upstream and 1 kb downstream of the TSS, highlighting the biologically relevant region for promoter modeling.

Based on this biological evidence, we focused on modeling promoter sequences in windows extending several kilobases upstream and downstream of the TSS. This design allows us to capture both proximal promoter elements and longer-range regulatory signals.

Appendix C. Model Metrics

There are the summary of model metrics used to evaluate the fine-tuning of the DFMs used.

$$\text{Accuracy} = \frac{TP + TN}{TP + TN + FP + FN}$$

$$\text{Precision} = \frac{TP}{TP + FP}$$

$$\text{Recall} = \frac{TP}{TP + FN}$$

$$F1 = \frac{2 \times \text{Precision} \times \text{Recall}}{\text{Precision} + \text{Recall}}$$

$$MCC = \frac{TP \times TN - FP \times FN}{\sqrt{(TP + FP)(TP + FN)(TN + FP)(TN + FN)}}$$

Appendix D. Fine tuning of DNABERT2, Nucleotide Transformer and GENA-LM best models table

The table below extends Figure. 1 by reporting per–tissue results across sequence lengths (2,000, 3,000, and 4,000 bp) and learning–rate settings (see the `Run` column). The best performance was obtained when using Wide or Null as the negative class. *Wide* (widespread) promoters typically correspond to housekeeping or basal regulatory functions, whereas *Null* denotes minimal expression in adult normal tissues. These observations motivated our choice of Wide/Null as the negative set for fine-tuning, to better capture the regulatory logic of TSp promoter regions.

Unless otherwise specified, models were trained with: weight decay $= 1\times10^{-2}$, warmup steps = 10, epochs = 10, and gradient accumulation steps = 1. We used sequence length in $\{2k, 3k, 4k\}$ bp and learning rates in a small grid covering $[5\times10^{-6}, 7\times10^{-6}]$ and $[2\times10^{-5}, 3\times10^{-5}]$; the exact setting for each run is indicated in the table.

Table 2: Performance comparison of DNABERT2, GENA-LM, and NT (500m--1000G) models across tissues and sequence lengths. Metrics include Precision, Recall, F1-score, MCC, and Accuracy.

Length (bp)	Tissue	TSp site	Run (LR)	Precision	Recall	F_1	MCC	Accuracy	Model
2000	Brain	null	lr5e-6	80.26	80.23	80.22	60.49	80.23	DNABERT2
3000	Brain	null	lr3e-6	81.21	81.19	81.19	62.40	81.19	DNABERT2
4000	Brain	null	lr3e-6	80.07	80.06	80.06	60.13	80.06	DNABERT2
2000	Liver	wide	lr5e-6	79.57	78.10	77.82	57.65	78.10	DNABERT2
3000	Liver	wide	lr5e-6	84.77	84.71	84.70	69.48	84.71	DNABERT2
4000	Liver	wide	lr7e-6	80.83	80.58	80.54	61.41	80.58	DNABERT2
2000	Testis	wide	lr7e-6	76.73	76.27	76.16	52.99	76.27	DNABERT2
3000	Testis	wide	lr7e-6	76.97	75.83	75.56	52.79	75.83	DNABERT2
4000	Testis	wide	lr3e-5	77.37	77.20	77.16	54.57	77.20	DNABERT2
2000	Muscle	null	lr3e-6	67.97	67.31	67.00	35.27	67.31	DNABERT2
3000	Muscle	null	lr7e-6	67.97	67.31	67.00	35.27	67.31	DNABERT2
4000	Muscle	null	lr5e-6	71.15	71.15	71.15	42.31	71.15	DNABERT2
2000	Spleen	null	lr7e-6	74.48	73.96	73.82	48.43	73.96	DNABERT2
3000	Spleen	low	lr7e-6	75.05	72.92	72.33	47.92	72.92	DNABERT2
4000	Spleen	null	lr5e-6	78.31	77.08	76.83	55.38	77.08	DNABERT2
2000	Brain	null	lr2e-5	76.26	82.64	79.32	57.11	78.46	GENA-LM
3000	Brain	null	lr2e-5	79.08	82.64	80.82	60.83	80.39	GENA-LM
4000	Brain	null	lr5e-6	78.41	88.75	83.26	64.87	82.15	GENA-LM
2000	Liver	wide	lr5e-6	78.20	85.95	81.89	62.29	80.99	GENA-LM
3000	Liver	tenh	lr5e-6	72.78	95.04	82.44	62.50	79.75	GENA-LM
4000	Liver	wide	lr5e-6	75.40	78.51	76.92	52.94	76.45	GENA-LM
2000	Testis	tenh	lr2e-5	75.86	74.52	75.19	50.82	75.41	GENA-LM
3000	Testis	wide	lr5e-6	77.70	75.83	76.75	54.08	77.03	GENA-LM
4000	Testis	tenh	lr2e-5	72.47	79.68	75.91	49.67	74.71	GENA-LM
2000	Muscle	low	lr5e-6	54.02	90.38	67.63	18.20	56.73	GENA-LM
3000	Muscle	low	lr3e-4	50.00	100.00	66.67	0.00	50.00	GENA-LM
4000	Muscle	low	lr2e-5	52.50	40.38	45.65	3.95	51.92	GENA-LM
2000	Spleen	low	lr2e-5	75.61	64.58	69.66	44.22	71.88	GENA-LM
3000	Spleen	low	lr5e-6	80.00	66.67	72.73	50.71	75.00	GENA-LM
4000	Spleen	low	lr2e-5	57.63	70.83	63.55	19.26	59.38	GENA-LM
2000	Brain	low	lr5e-6	68.14	66.98	66.44	35.10	66.98	NT (500m–1000G)
3000	Brain	null	lr5e-6	71.36	71.21	71.16	42.57	71.21	NT (500m–1000G)
4000	Brain	null	lr5e-6	74.39	74.04	73.95	48.42	74.04	NT (500m–1000G)
2000	Liver	low	lr2e-5	62.25	62.22	62.20	24.47	62.22	NT (500m–1000G)
3000	Liver	null	lr2e-5	59.63	59.55	59.47	19.18	59.55	NT (500m–1000G)
4000	Liver	null	lr2e-5	62.60	62.50	62.42	25.10	62.50	NT (500m–1000G)
2000	Muscle	low	lr3e-4	52.44	51.92	49.22	4.34	51.92	NT (500m–1000G)
3000	Muscle	low	lr3e-4	50.00	50.00	49.33	0.00	50.00	NT (500m–1000G)
4000	Muscle	low	lr2e-5	53.99	53.85	53.42	7.84	53.85	NT (500m–1000G)
2000	Spleen	low	lr2e-5	60.43	60.42	60.40	20.85	60.42	NT (500m–1000G)
3000	Spleen	low	lr2e-5	65.80	65.62	65.53	31.42	65.62	NT (500m–1000G)
4000	Spleen	low	lr2e-5	55.21	55.21	55.20	10.42	55.21	NT (500m–1000G)
2000	Testis	low	lr5e-6	53.44	53.25	52.60	6.69	53.25	NT (500m–1000G)
3000	Testis	low	lr5e-6	57.04	56.25	54.99	13.27	56.25	NT (500m–1000G)
4000	Testis	low	lr5e-6	59.15	59.00	58.83	18.15	59.00	NT (500m–1000G)

Appendix E. Other approaches to build better models did not increase the model performance

E.1. Motivation

CpG and non-CpG promoter profiles differ in their sequence and regulatory properties, motivating us to test whether separating these groups could improve model performance (Landolin et al., 2010; Vavouri and Lehner, 2012). We therefore hypothesized that pretraining two `DNABERT2` models—one on CpG promoters and another on non-CpG promoters—followed by fine-tuning might better capture their distinct features. However, this split introduced strong tissue imbalances; for example, in liver the CpG subset is small (≈ 250 promoters) compared with non-CpG. Consequently, the effective sample sizes were much smaller than in our original setup, which likely explains the poor results we observed. It is also known that, in general, TSp regions contain more non-CpG promoters than other expression groups. These findings are consistent with prior reports linking Non-CpG promoters to TSp genes and CpG-rich promoters to housekeeping genes that are constitutively active across tissues (Saxonov et al., 2006; Vavouri and Lehner, 2012).

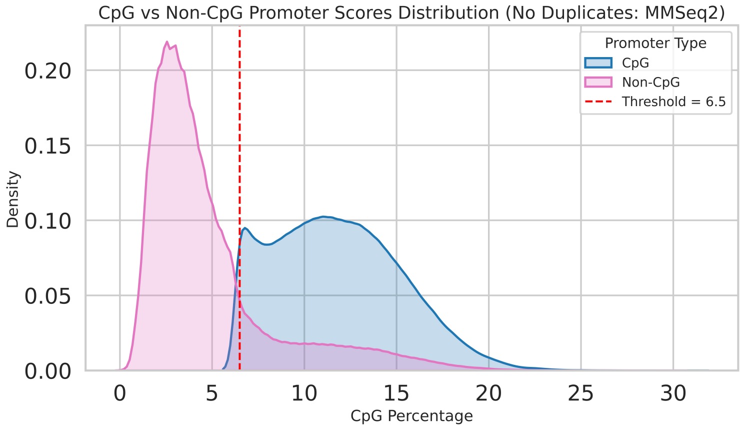

Figure 2: 201bp windows along 6k length around TSS (5k, 1k, TSS) CpG score distribution across the dataset used for pretraining.

E.2. Pretraining and Fine-Tuning

We pretrained models using 6 kb sequences centered around transcription start sites (TSS) from 30 species, including primates (e.g., *C. syrichta*, *A. nancymaae*, *G. gorilla*, *H. sapiens*, *M. mulatta*, *M. nemestrina*, *N. leucogenys*, *O. garnettii*, *P. abelii*, *P. anubis*, *P. paniscus*, *P. troglodytes*, *R. bieti*, *R. roxellana*, *R. norvegicus*, *S. boliviensis*) and rodents (e.g., *H. griseus*, *C. lanigera*, *I. tridecemlineatus*, *J. jaculus*, *M. murinus*, *M. caroli*, *M. musculus*, *M. pahari*, *M. spicilegus*, *M. spretus*, *N. galili*, *P. bairdii*, *R. norvegicus*, *M. fascicularis*), obtained via `biomaRt` (v2.60.1) (Smedley et al., 2009). However, during fine-tuning, the models did not show substantial improvement, as summarized in Table 3. For fine-tuning, we further stratified promoters into CpG-rich and non-CpG groups using a 6.5% CpG-content cutoff computed over sliding 201 bp windows across the TSS-centered region, but this splitting did not yield significant performance gains.

Table 3: Results after further pretraining on CpG vs. non-CpG promoters, followed by fine-tuning.

Experiment	TSp site	TransTEx group	Length	LR			F_1	MCC	Accuracy
Non-CpG Further Pretrained Model									
	Brain	Null	2000	5e-6	82.95	82.89	82.83	65.84	82.83
	Liver	Wide	3000	3e-6	82.12	82.05	81.94	64.17	81.94
	Liver	Wide	3000	5e-6	81.61	81.57	81.48	63.18	81.48
	Liver	Wide	3000	7e-6	81.47	81.49	81.48	62.96	81.48
	Brain	Null	2000	7e-6	81.14	81.08	81.02	62.22	81.02
CpG Further Pretrained Model									
	Brain	Null	4000	5e-6	79.10	78.60	78.77	57.70	79.15
	Brain	Null	4000	7e-6	77.65	77.17	77.33	54.82	77.74
	Brain	Wide	4000	7e-6	76.56	76.50	76.52	53.05	76.68

Appendix F. Brain Models to understand promoter biology

F.1. Model A for Brain

This shows that Model A shows a more evenly distribution of the identified motifs around TSS with peaks around the core and proximal promoter regions for TF Zic3 that is uniquely present in this model and not Model B. This shows our model is able to capture general promoter as well as TSp promoter enriched tokens which imply critical role in biology.

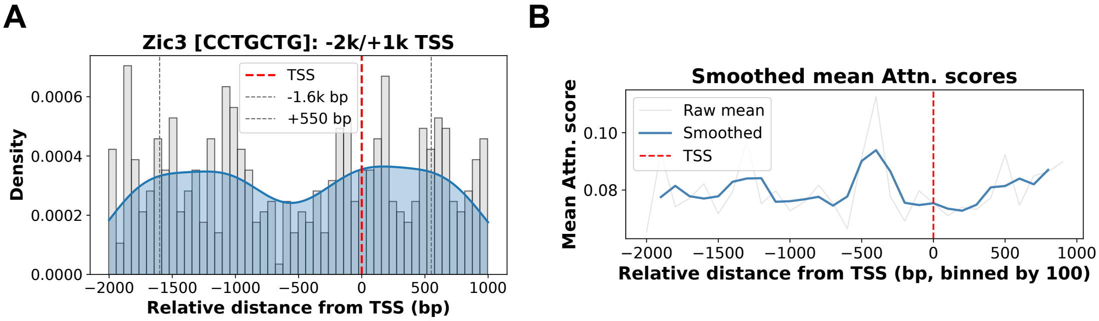

Figure 3: Detailed analysis of the Zic3 transcription factor motif. (**A**) Density of motif occurrences relative to the transcription start site (TSS), with the TSS (red dashed line), upstream −1.6 kb, and downstream +550 bp indicated. (**B**) Smoothed mean attention scores relative to the TSS, highlighting enrichment near promoter regions.

F.2. Model B for Brain

This shows that Model B shows a more evenly distribution of the identified motifs around TSS with peaks around the core and proximal promoter regions for TF SP1 that is ubiquitously present TF in both Models A and B. This shows our model is able to capture global brain promoter logic.

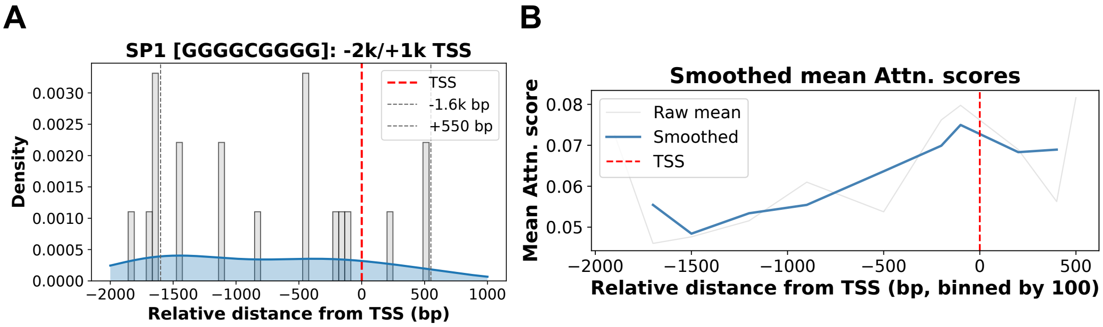

Figure 4: Detailed analysis of the SP1 transcription factor motif. (**A**) Density of SP1 TF motif occurrences relative to TSS (red), upstream −1.6 kb, and downstream +550 bp indicated. (**B**) Smoothed mean attention scores relative to the TSS, highlighting enrichment regions.

F.3. Model A and Model B clinically relevant TFs

We summarize the clinically relevant motifs for brain from KNOCK-TF 2.0 and TF-marker for brain specific TFs.

Table 4: Representative hits from KnockTF 2.0 (Feng et al., 2024) showing enrichment of "GCACGT" motif in glioblastoma and neuroblastoma cell lines.

Query_ID	Target_ID	Offset	p-value	E-value	q-value	Overlap	TF_name	Tissue Type	Cell line
GCACGT	MA1493.1	1	7.46E-05	0.175107	0.0173884	6	HES6	Brain	A172 (glioblastoma)
GCACGT	MA1493.1	1	7.46E-05	0.175107	0.0173884	6	HES6	Brain	LN405 (glioblastoma)
GCACGT	MA0147.4	0	2.37E-04	0.556926	0.0291687	6	MYC	Brain	BE(2)-C (neuroblastoma)
GCACGT	MA0147.4	0	2.37E-04	0.556926	0.0291687	6	MYC	Brain	NB1691 (neuroblastoma)
GCACGT	MA0147.4	0	2.37E-04	0.556926	0.0291687	6	MYC	Brain	BE(2)-C (neuroblastoma)
GCACGT	MA0147.4	0	2.37E-04	0.556926	0.0291687	6	MYC	Brain	BE(2)-C (neuroblastoma)

Table 5: Representative TF-Marker (Xu et al., 2022) hits showing motif mapping to *SP1* and *HIF1A* in brain cell types.

Query_ID	Target_ID	Offset	p-value	q-value	Overlap	TF_name	Tissue Type	Cell type	Gene Name
RGYCWVGGGGVGGGG	MA0079.5	-6	3.18E-06	0.00293195	9	SP1	Brain	Nerve cell (normal)	SP1
GCACGT	MA1106.2	0	2.63E-05	0.0173884	6	HIF1A	Brain	Glial cell (cancer)	HIF1A

Appendix G. Testis-specific regulatory language

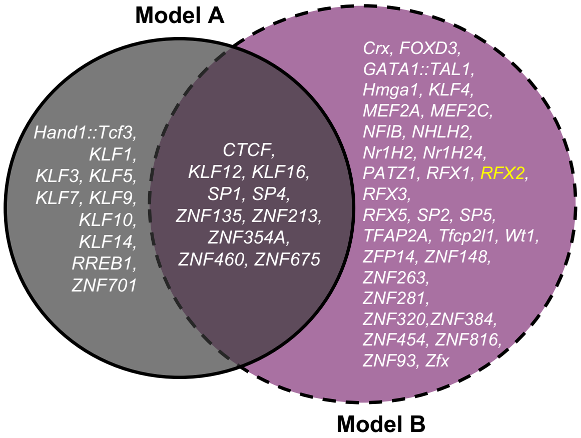

Figure 5: Overlap of top significant motifs in brain identified across Models A and B found among JASPAR hits. The same framework was applied to testis data to reveal transcription factors uniquely enriched in testis-specific promoters.

Appendix H. High-Attention Regions Reveal Significant Global TSp Regulatory Motifs

H.1. Model B for all TSp promoters in human

We summarize the significant top motifs identified from the JASPAR database among the significant motifs identified in all TSp promoters in human. We identify rich "CG" repeat.

Table 6: Representative significant motifs identified from enrichment analysis from the JASPAR databse (Rauluseviciute et al., 2024).

Query_ID	Target_ID	Offset	p-value	q-value	Overlap	Query_consensus	Target_consensus	Orientation	TF_name	Class	Family
TGCAGCTGTG	MA0521.3	-2	4.50E-07	0.00052	6	TGCAGCTGTG	CAGCTG	+	Tcf12	bHLH	E2A
TGCAGCTGTG	MA0633.3	-2	6.24E-06	0.00206	6	TGCAGCTGTG	CAGCTG	+	Twist2	bHLH	Tal-related
TGCAGCTGTG	MA1993.2	-2	6.24E-06	0.00206	6	TGCAGCTGTG	CAGCTG	+	Neurod2	bHLH	Tal-related
TGCAGCTGTG	MA1997.2	-2	6.24E-06	0.00206	6	TGCAGCTGTG	CAGCTG	+	Olig2	bHLH	Tal-related
GGAGGCYGAGGYDGS	MA1596.1	4	1.71E-06	0.00456	12	GGAGGCCGAGGTGGG	CTCGGGAGGCTGAGGC	-	ZNF460	C2H2	Zinc fingers
GGGAGGCWGAA	MA1965.2	0	1.19E-06	0.00559	6	GGGAGGCAGAA	GGGAGG	-	SP5	C2H2	Kruppel-related
GRTGRGGRAAAGGRGGVWGG	MA1723.2	-2	1.21E-06	0.00566	18	GATGAGGGAAAGGAGGAAGG	GGTGGGCAGGGAGGAAGCAG	+	PRDM9	C2H2	Multiple ZFs
TGCAGCTGTG	MA1635.2	-2	2.17E-05	0.00626	6	TGCAGCTGTG	CAGCTG	+	BHLHE22	bHLH	Tal-related
CCGGGAGGYGGAG	MA1596.1	1	1.71E-06	0.00801	13	CCGGGAGGCGGAG	CTCGGGAGGCTGAGGC	-	ZNF460	C2H2	Zinc fingers
GGGGGCCC	MA1548.2	0	4.98E-06	0.01154	8	GGGGGCCC	GGGGGCCC	-	PLAGL2	C2H2	Zinc fingers
TGCAGCTGTG	MA1641.2	-1	6.22E-05	0.01487	8	TGCAGCTGTG	ACAGCTGT	+	MYF5	bHLH	MyoD/ASC
TGCAGCTGTG	MA1472.3	-1	6.76E-05	0.01487	8	TGCAGCTGTG	ACAGCTGT	+	Bhlha15	bHLH	Tal-related
TGCAGCTGTG	MA0048.3	-1	6.39E-05	0.01487	9	TGCAGCTGTG	GCAGCTGCG	-	NHLH1	bHLH	Tal-related
TGCAGCTGTG	MA0500.3	-1	8.00E-05	0.01546	8	TGCAGCTGTG	GCAGCTGC	+	MYOG	bHLH	MyoD/ASC
DYYYDGYTAAWWTWTKTTTT	MA1978.2	0	1.42E-05	0.01659	20	TTCTAGCTAATTTTTTTTTT	AATTAGTCCATTTATATTTA	-	ZNF354A	C2H2	Zinc fingers
WRGGAGGSGMYGGGCYGG	MA1522.2	-1	3.94E-06	0.01846	8	AAGGAGGGGATGGGCTGG	GGGAGGGG	-	MAZ	C2H2	Zinc fingers
TGCAGCTGTG	MA1619.2	-1	1.25E-04	0.02219	8	TGCAGCTGTG	ACAGCTGT	+	Ptf1A	bHLH	Tal-related
GGGAGGCWGAA	MA1596.1	3	9.49E-06	0.02228	11	GGGAGGCAGAA	CTCGGGAGGCTGAGGC	-	ZNF460	C2H2	Zinc fingers
CCGGGAGGYGGAG	MA1587.1	0	1.06E-05	0.02490	13	CCGGGAGGCGGAG	TCAGGAGGTCGAGG	-	ZNF135	C2H2	Zinc fingers
WCCAGCCTGGGCRAC	MA2121.1	0	5.69E-06	0.02671	12	TCCAGCCTGGGCAAC	CGCAGCCTGGGC	-	ZNF213	C2H2	Zinc fingers
AGCTAGACCTCCCTGCTGC	MA1628.2	-11	6.13E-06	0.02874	7	AGCTAGACCTCCCTGCTGC	CCTGCTG	-	Zic1::Zic2	C2H2	Zinc fingers
AGCTAGACCTCCCTGCTGC	MA0697.3	-11	1.23E-05	0.02884	7	AGCTAGACCTCCCTGCTGC	CCTGCTG	-	Zic3	C2H2	Zinc fingers
GGAGGCYGAGGYDGS	MA1972.1	0	8.56E-05	0.03985	15	GGAGGCCGAGGTGGG	GGAGGCACTGGAATG	+	ZFP14	C2H2	Zinc fingers
GGAGGCYGAGGYDGS	MA1723.2	0	7.91E-05	0.03985	15	GGAGGCCGAGGTGGG	GGTGGGCAGGGAGGAAGCAG	+	PRDM9	C2H2	Zinc fingers
TTTTTKTWTTTTTWK	MA1125.2	-5	6.49E-05	0.04132	8	TTTTTTTTTTTTTTG	TTTTTTTT	-	ZNF384	C2H2	Zinc fingers
GGAGGCYGAGGYDGS	MA1587.1	3	9.98E-05	0.04223	11	GGAGGCCGAGGTGGG	TCAGGAGGTCGAGG	-	ZNF135	C2H2	Zinc fingers
TGCAGCTGTG	MA0499.3	-1	2.89E-04	0.04456	9	TGCAGCTGTG	GCACCTGTC	+	MYOD1	bHLH	MyoD/ASC
CAGCWMYTYGGGAGG	MA1965.2	-9	9.80E-06	0.04599	6	CAGCTACTTGGGAGG	GGGAGG	-	SP5	C2H2	Kruppel-related

References

Chenchen Feng, Chao Song, Shuang Song, Guorui Zhang, Mingxue Yin, Yuexin Zhang, Fengcui Qian, Qiuyu Wang, Maozu Guo, and Chunquan Li. Knocktf 2.0: a comprehensive gene expression profile database with knockdown/knockout of transcription (co-)factors in multiple species. *Nucleic Acids Research*, 52 (D1):D183–D193, January 2024. doi: 10.1093/nar/gkad1016.

Ilias Georgakopoulos-Soares, Chengyu Deng, Vikram Agarwal, Candace SY Chan, Jingjing Zhao, Fumitaka Inoue, and Nadav Ahituv. Transcription factor binding site orientation and order are major drivers of gene regulatory activity. *Nature communications*, 14(1):2333, 2023.

Jane M. Landolin, David S. Johnson, Nathan D. Trinklein, Shelly F. Aldred, Catherine Medina, Hennady Shulha, Zhiping Weng, and Richard M. Myers. Sequence features that drive human promoter function and tissue specificity. *Genome Research*, 20(7):890–898, 2010. doi: 10.1101/gr.100370.109.

Ieva Rauluseviciute, Roser Riudavets-Puig, Roselyne Blanc-Mathieu, Jaime A. Castro-Mondragon, Krisztian Ferenc, Vineet Kumar, Rediet B. Lemma, Jerome Lucas, Jeanne Chèneby, Damir Baranasic, Aziz Khan, Oriol Fornes, Sveinung Gundersen, Marianne Johansen, Eivind Hovig, Boris Lenhard, Albin Sandelin, Wyeth W. Wasserman, François Parcy, and Anthony Mathelier. Jaspar 2024: 20th anniversary of the open-access database of transcription factor binding profiles. *Nucleic Acids Research*, 52(D1):D174–D182, 2024. doi: 10.1093/nar/gkad1059.

Serge Saxonov, Paul Berg, and Douglas L. Brutlag. A genome-wide analysis of cpg dinucleotides in the human genome distinguishes two distinct classes of promoters. *Proceedings of the National Academy of Sciences*, 103(5):1412–1417, 2006. doi: 10.1073/pnas.0510310103.

Damian Smedley, Syed Haider, Benoit Ballester, Richard Holland, Darin London, Gudmundur Thorisson, and Arek Kasprzyk. Biomart–biological queries made easy. *BMC Genomics*, 10(1):22, 2009. doi: 10.1186/1471-2164-10-22.

Kannan Tharakaraman, Olivier Bodenreider, David Landsman, John L Spouge, and Leonardo Mariño-Ramírez. The biological function of some human transcription factor binding motifs varies with position relative to the transcription start site. *Nucleic acids research*, 36(8):2777–2786, 2008.

Tanya Vavouri and Ben Lehner. Human genes with cpg island promoters have a distinct transcription-associated chromatin organization. *Genome Biology*, 13(11):R110, 2012. doi: 10.1186/gb-2012-13-11-r110.

Srinivas Veerla and Mattias Höglund. Analysis of promoter regions of co-expressed genes identified by microarray analysis. *BMC bioinformatics*, 7(1):384, 2006.

Mingcong Xu, Xuefeng Bai, Bo Ai, Guorui Zhang, Chao Song, Jun Zhao, Yuezhu Wang, Ling Wei, Fengcui Qian, Yanyu Li, Xiaohong Zhou, Lihua Zhou, Yan Yang, Jie Chen, Jun Liu, Dan Shang, Xiaobo Wang, Ying Zhao, Xia Huang, Yifan Zheng, Jing Zhang, Qiuyu Wang, and Chunquan Li. Tf-marker: a comprehensive manually curated database for transcription factors and related markers in specific cell and tissue types in human. *Nucleic Acids Research*, 50(D1):D402–D412, January 2022. doi: 10.1093/nar/gkab1114.
